# From genetic data to kinship clarity: employing machine learning for detecting incestuous relations

**DOI:** 10.3389/fgene.2025.1578581

**Published:** 2025-06-02

**Authors:** Dejan Šorgić, Aleksandra Stefanović, Mladen Popović, Dušan Keckarević

**Affiliations:** ^1^ Department of Paternity Identification, Biological and Other Traces, Institute of Forensic Medicine, Niš, Serbia; ^2^ Department of English, Faculty of Philosophy, University of Niš, Niš, Serbia; ^3^ Department of Biochemistry and Molecular Biology, Faculty of Biology, University of Belgrade, Belgrade, Serbia

**Keywords:** machine learning, forensic genetics, kinship analysis, incest detection, STR profiling

## Abstract

**Introduction:**

The aim of the study was to develop a predictive model based on STR profiles of mothers and children for the detection of incestuous conception.

**Methods:**

Based on allele frequency data from the USA and Saudi Arabia, STR profiles were generated and used to simulate offspring profiles corresponding to father-child and brother-sister incest scenarios. Model training and evaluation were performed using the STR profiles of the mother and child. In addition to the baseline model, we examined its performance under a one-step mutation model, as well as its ability to detect incestuous relationships based solely on the child's STR profile. Several machine learning algorithms and neural networks were tested for classification accuracy.

**Results:**

The CatBoost algorithm performed best in the binary classification of Normal Paternity vs. Incest Kinship. For the USA, we achieved the following results: 96.94% for 29 markers and 95% for 21 markers. The same accuracy was obtained with a single-step mutation, while prediction based on child profiles exclusively yielded an accuracy of 90.37% in the U.S. population. When analysing profiles from Saudi Arabia and modified Saudi frequencies, an accuracy of 94% was achieved.

**Discussion:**

It was established that population structure does not affect the model's accuracy and that it can be applied even in isolated populations.

## 1 Introduction

Incest is an extreme form of inbreeding that can lead to serious genetic consequences. While inbreeding is a broader genetic concept that refers to reproduction between biologically related individuals, incest specifically denotes close kin matings that are also socially or legally prohibited (Alvarez et al., 2009; Habicht et al., 2015; Rudan and Campbell, 2004). One of the best-known historical examples is the Spanish Habsburg dynasty, which became extinct due to long-term consanguineous marriages that resulted in increased homozygosity and pronounced inbreeding depression (Alvarez et al., 2009). Such marriages are not new, as documented in the example of ancient Egypt, where such unions were encouraged within royal families. Physical-anthropological analysis of the mummified pharaohs indicated a lower degree of phenotypic variation compared to that observed in the general population. As body height is inherited polygenetically, it can be used as one of the indicators of inbreeding, especially in cases where DNA analysis is not feasible (Habicht et al., 2015). In addition to early developmental outcomes, inbreeding may also exert substantial effects on human health later in life. Inbred individuals may carry a greater burden of rare, recessive variants that can contribute to late-onset diseases such as cardiovascular disorders, cancer, and psychiatric conditions, thereby potentially reducing life expectancy (Rudan and Campbell, 2004).

To better understand and detect incest, the field of hereditary analysis has evolved significantly since the time of Gregor Mendel. Historically, one of the first methods of hereditary analysis was based on the identification of blood groups or allelic products. Although advanced for its time, these methods had extremely limited specificity and sensitivity. The development of highly heterozygous loci and the determination of probabilities obtained by comparing maternal and offspring phenotypes have significantly improved the accuracy of testing in cases of incest. The importance of these results is further emphasized by the fact that increased homozygosity due to incest may occur, but is not always pronounced, especially after a single generation of inbreeding (Wenk et al., 1994).

Research based on the analysis of VNTRs (Variable Number Tandem Repeats) found that offspring from incestuous relationships have higher homozygosity compared to offspring resulting from random mating (Corach et al., 2003). For a time, this method of analysis was even mandatory in the United States of America before adoption, especially in cases in which the mother was not available for testing or was not known (Wenk et al., 1994) Higher homozygosity rates were also confirmed by human leukocyte antigen (HLA) testing, which showed higher exclusion rates in cases of suspected incest (Houtz et al., 1982).

Congenital malformation rates worldwide range between 3% and 5%, while in Arab populations, they are above 7%. Some of the diseases that occur as a result of autosomal recessive disorders are Bardet-Biedl syndrome, Meckel-Gruber syndrome, osteopetrosis, and congenital chloride diarrhea (Tadmouri et al., 2009) The increased homozygosity observed in incestuous offspring emphasizes the importance of genetic screening and analysis in cases where an incest is suspected. Another testing method that has proven to be effective in complex consanguinity and incest cases is X chromosome analysis, as it provides additional information about inheritance that complements classical autosomal STR markers. This method is particularly useful for distinguishing between incestuous relationships involving the father and those involving the brother, since a father and son never share the same X chromosome (Garcia et al., 2022) By introducing single nucleotide polymorphisms into the procedure (SNPs), this method was able to further improve the accuracy of kinship analysis, including in cases of suspected incest (Fan et al., 2013; Dario et al., 2011).

The use of machine learning (ML) in genetics has the ever augmenting potential to facilitate the analysis of genetic data, which, with the introduction of new methods and technologies, has become more complex and demanding to analyse. Machine learning methods have already demonstrated significant advantages over classical statistical approaches. For instance, a study by Skowronski and associates used supervised machine learning algorithms in the context of classification within highly similar genetic populations of plants (96.88% similarity), and demonstrating the superiority of ML models over classic statistical models (Skowronski et al., 2021).

The aim of our research is to extend the application of machine learning algorithms to the prediction of incest. The model selected and used for that purpose is CatBoost (Category Boost), which is based on gradient-boosted decision trees (GBDT) for categorical data, developed by Yandex. The model features Ordered Target Statistics, which prevents target leakage by ensuring that the data category encoding does not use its own target value during training, and Ordered Boosting, which reduces overfitting by using random permutations of the data to build trees (Hancock and Khoshgoftaar, 2020).

The effectiveness of CatBoost has been confirmed by several previous studies in the field of genetics. Regarding the specificities of its use, in one study CatBoost was used to predict genotype matching between patient-derived xenografts (PDX) and original lung tumours (He et al., 2022). CatBoost has proven itself to be extremely successful in classifying genes associated with Alzheimer’s disease, achieving an accuracy rate of 96%^.^ (Shukla and Singh, 2023) Finally, CatBoost was also highly effective in classifying brain tumours based on gene expression, with an accuracy rate of 91% (Almars et al., 2021).

We aim to expand this body of work by presenting a novel predictive model based on the CatBoost algorithm for detecting incestuous relationships. To achieve this, we first generated DNA profiles corresponding to different relationship categories: normal parent-child profiles, sibling profiles, father-child cross-mating profiles, and sibling cross-mating profiles. The performance of the model was examined for different populations, using corresponding allele frequencies as input variables, including the single-step mutation scenarios. In order to better understand and improve the model, we monitored homozygosity, as well as the appearance of identical heterozygous loci in different categories of cross-mating. The results proved to be consistent for different scenarios, even in situations in which incest prediction was made utilizing solely the profiles of children.

## 2 Methodology

### 2.1 Data collection

To investigate whether population structure affects model performance, we decided to focus on two genetically and socially distinct populations. For the general population, we used allele frequencies from the United States, which is characterized by high genetic diversity, while Saudi Arabia was selected as a contrast, due to its lower genetic diversity resulting from frequent endogamous marriages.

For the general population, the frequencies were obtained from a previously published sample of 1,036 unrelated subjects from the USA, representing four different ethnic groups (African Americans, Caucasians, Asians, Hispanics) (Steffen et al., 2017). The loci included in the database are: CSF1PO, D10S1248, D12S391, D13S317, D16S539, D18S51, D19S433, D1S1656, D21S11, D22S1045, D2S1338, D2S441, D3S1358, D5S818, D6S1043, D7S820, D8S1179, F13A01, F13B, FESFPS, FGA, LPL, Penta C, Penta D, Penta E, SE33, TH01, TPOX, and vWA. (See Supplementary Table S1, General Population Data).

Allele frequencies of subjects from Saudi Arabia were obtained from a separate study. The authors of the study in question obtained allele frequencies for the population and observed an increased rate of homozygosity, which was argued to be a consequence of higher consanguinity rates in Saudi Arabia (Khubrani et al., 2019). The initial database contained the following loci: D3S1358, vWA, D16S539, CSF1PO, TPOX, D8S1179, D21S11, D18S51, D2S441, D19S433, TH01, FGA, D22S1045, D5S818, D13S317, D7S820, SE33, D10S1248, D1S1656, D12S391, and D2S1338. This database was further modified and all alleles with frequencies of 0.001 and 0.002 were deleted. As a result, the final database contained values for 215 alleles, a reduction from the original 263 alleles present in the initial database. After that, normalisation was performed for the remaining alleles in order to obtain a uniform distribution of the remaining alleles. (See Supplementary Table S2, Saudi Arabia Population Data; Supplementary Table S3, Saudi Arabia Modified Data).

### 2.2 Profile generation and crossings

The initial script was created in Python and was used to generate 50,000 STR profiles. Each individual genetic profile was composed of markers, represented by two columns, each containing data for a single allele. Columns with the suffix “1” contain values for the smaller alleles and columns with the suffix “2” contain values for the larger alleles.

The next step of our procedure randomly matched two individual profiles, yielding a single unified combination of parental profiles. After that, four children’s profiles were generated using the parent profiles as a basis, yielding 100,000 child profiles in total. After reorganising these profiles, we obtained 200,000 parent-child profile combinations belonging to the category of Normal Paternity.

The same procedure was applied to obtain profiles that belong to the category of Incestuous Paternity. This was done by pairing parent profiles with their respective child profiles to generate offspring profiles. The procedure yielded 200,000 parent-child profiles belonging to the Incestuous Paternity category.

Further, the previously generated child profiles (of normal parentage) were reorganised in a way to obtain 150,000 unique combinations of two profiles containing a single brother and a sister, belonging to the Normal Siblings category.

Finally, the normal sibling combined profiles were used to generate incestuous offspring profiles. These offspring profiles were joined with the sibling parent profiles and the procedure in question yielded 150,000 unique profiles, belonging to the Incestuous Siblings category.

All three databases described in 2.1 were formed in this way. As the profiles that belong to the general population were generated with 29 markers in total, the superfluous markers were excluded to match the markers in the general population database to the markers in the Saudi Arabian database.

### 2.3 One-Step Mutation Implementation

To account for genetic variations, we incorporated a one-step mutation rate into our model. A value of 10^–3^ was taken as the mutation rate (Butler et al., 2007), indicating that a mutation should occur at a rate of one mutation per 1,000 loci. As the markers were represented by two columns (one for each allele), the procedure changed the original allele value by 1, once every 2,000 alleles per column in the child profile database. (See Supplementary Table S4, Data with One Step Mutation).

### 2.4 Training and testing

Prediction models for the general population utilising 29 and 21 markers as input variables, as well as the basic and modified models for the Saudi Arabian population, were trained and tested using the initial database. The Normal Paternity category was composed of 200,000 parent-child profiles, while the Incest Kinship category (also containing 200,000 profiles) was assembled by randomly selecting 100,000 profiles from the Incest Sibs and Incest Paternity categories, combining them to form the aforementioned category.

The prediction model utilising solely children profiles, resulting from both normal and incestuous relationships, was trained and tested on a modified version of the initial dataset. The modified version was created by first extracting 21 loci (a reduction from the initial number of 29 loci). Next, all parental profiles were deleted (profiles with the prefix “1”) and the offspring profiles (profiles with the prefix “2”) were grouped into the following categories: Normal Paternity and Normal Sibs profiles were combined into the Normal Kinship category, while profiles from the Incest Sibs and Incest Paternity categories were grouped into the Incest Kinship category. In total, each category was composed of 350,000 profiles, resulting in a grand total of 700,000 profiles.

To prevent overfitting, regularisation and early stopping were implemented. The initial dataset was divided in the following way: 90% of the dataset was reserved for training, 5% was reserved for validation, and an additional 5% was reserved for testing. By following the described procedure, we obtained results that are not a consequence of the model’s excessive adaptation to the dataset.

**FIGURE 1:**
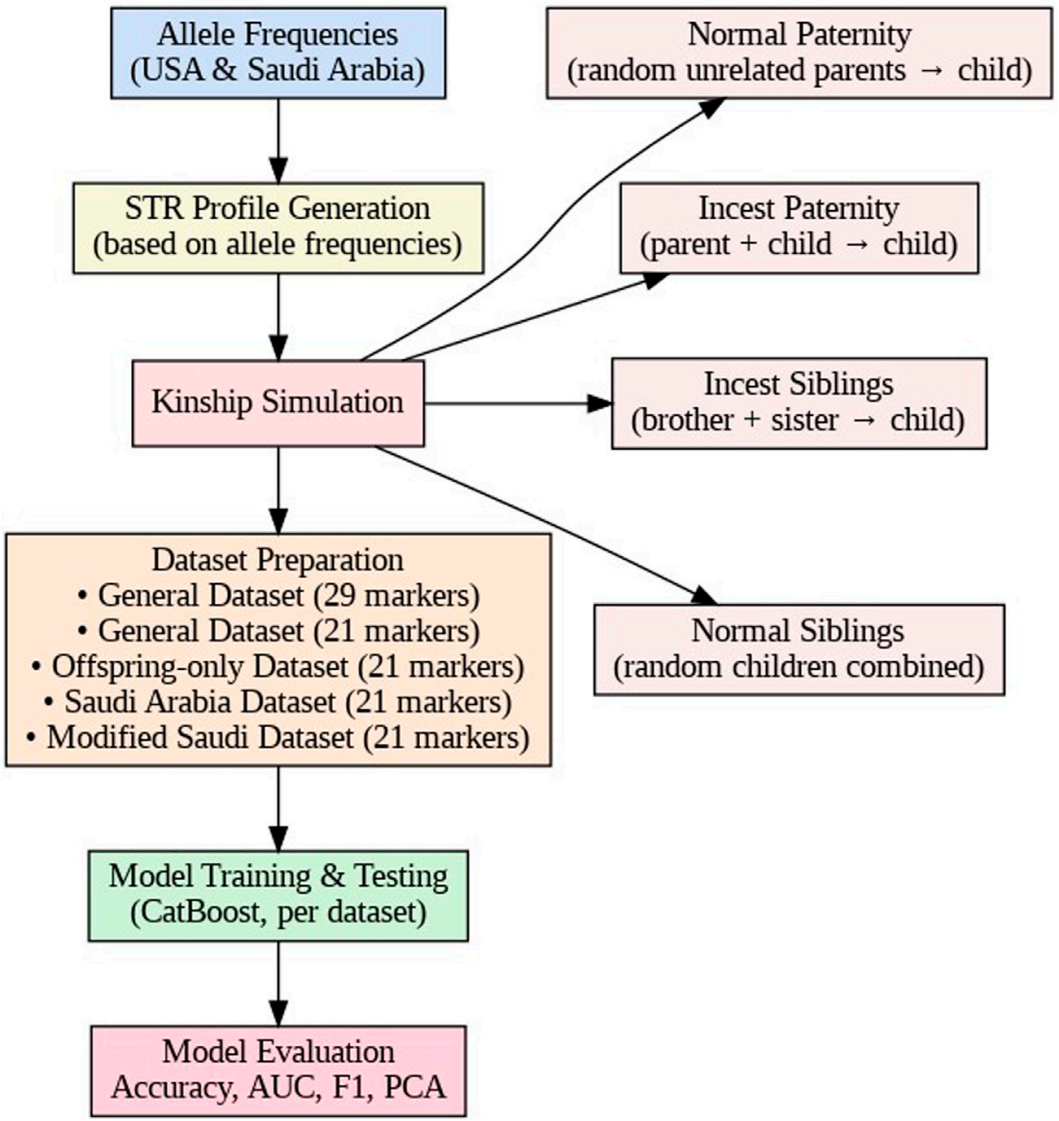
Workflow of STR-based kinship simulation, dataset preparation, and model evaluation.

To provide a visual summary of the procedure, an ideogram presented in Figure 1 was created to illustrate each step involved in the training and testing process.

## 3 Results

### 3.1 Machine learning models

Different machine learning algorithms and neural network assemblages were used in order to test initial suitability, of which the gradient boosting algorithms proved to be the most effective of the tested cohort. Among them, CatBoost performed best. Table 1 presents the tested algorithms and their performance in the binary classification of Normal Paternity and Incest Kinship. The model was trained and tested on a sample of 20,000 Normal Paternity profiles, 10,000 incestuous parent-child profiles and an additional 10,000 incestuous brother-sister profiles.

**TABLE 1:** Performance of various machine learning models in the binary classification of Normal Paternity vs. Incest Kinship.

Tested Algorithms	Accuracy (%)	Precision (%)	Recall	F1 Score	ROC AUC (%)
Random forest	78.1	76.97	80.2	78.55	85.85
Support vector machine	78.4	78.34	78.5	78.42	87.19
Naive Bayes	74.15	75.23	72	73.58	81.71
K-Nearest Neighbors	63.9	65.1	59.9	62.39	68
CatBoost	92.35	92.73	91.9	92.31	97.71
XGBoost	90.05	89.54	90.7	90.11	95.52
LightGBM	90.45	90.17	90.8	90.48	96.83
Adaboost	76.6	76.71	76.4	76.55	84.16
Gradient boosting	87.8	87.43	88.3	87.86	95.25
Extra trees	77.4	79.59	73.7	76.53	85.47
Multi-layer Perceptron	75.8	71.32	86.3	78.1	85.77

### 3.2 Model adaptation

After the initial run of the CatBoost model was completed, further refinement was performed to improve performance. The model’s loss was measured using the log loss function and the chosen evaluation metric was AUC (Area under the ROC curve). Subsequently, grid search was performed by iterating different parameters. For example, the number of iterations was varied [500, 1000], frequency was varied [0.01, 0.1] and tree depth was varied [6, 10].

The best parameters, identified through a comprehensive search, were the following: iterations = 1000, frequency = 0.1 and depth = 10, which were subsequently used in all presented models.

### 3.3 Model for the general population (29 Marker variant)

**FIGURE 2:**
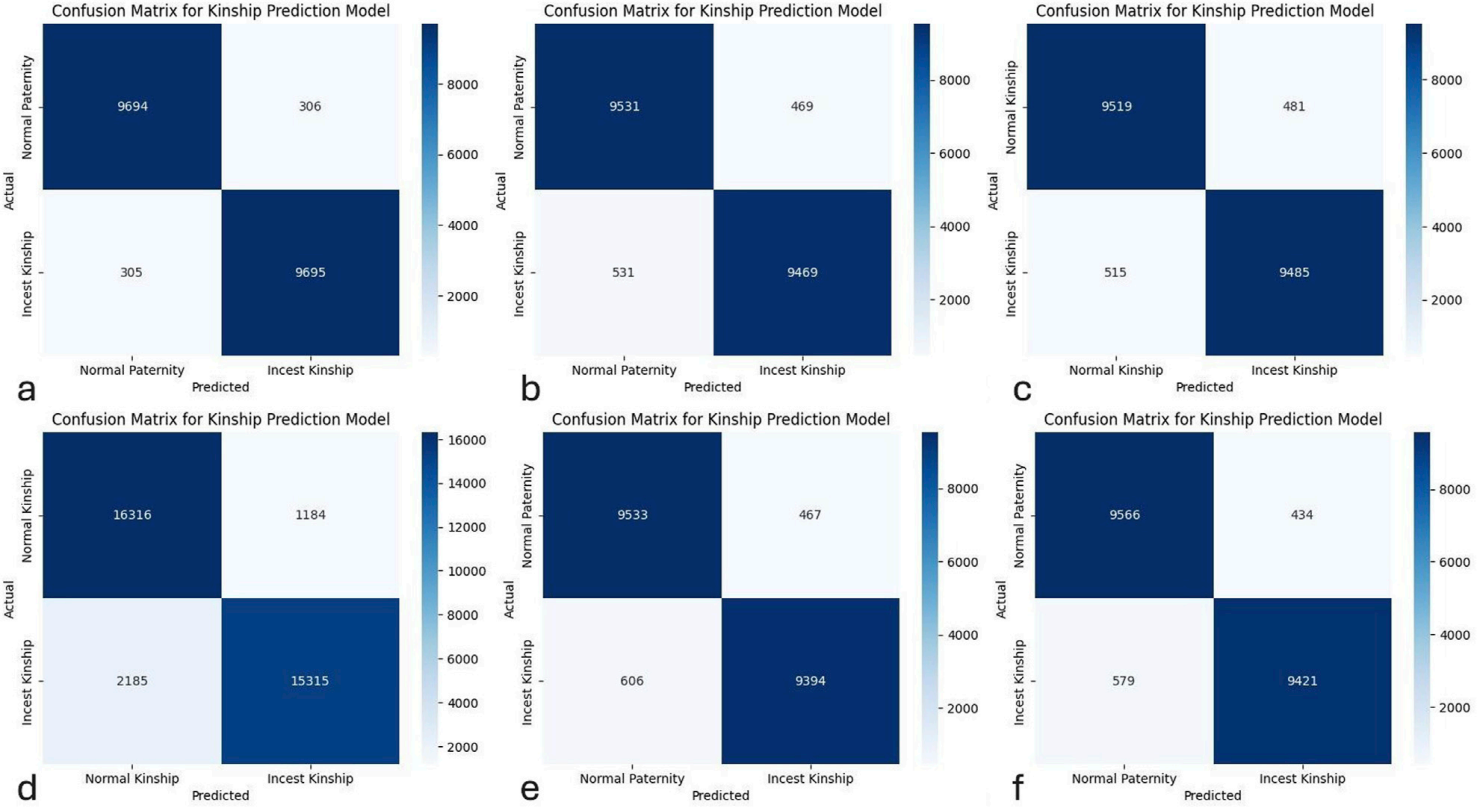
Confusion matrices for binary classification of normal and incest kinship profiles using CatBoost models. **(a)** General population − 29 markers; **(b)** General population − 21 markers; **(c)** General population with one-step mutation − 21 markers; **(d)** Offspring-only dataset − 21 markers; **(e)** Saudi Arabia dataset − 21 markers; **(f)** Modified Saudi Arabia dataset − 21 markers.

The binary classification model utilising 29 markers as input variables and the general population dataset for training and testing, resulted in an accuracy rate of 96.94%. The confusion matrix is presented in Figure 2a. The performance of the model across classes is as follows: 9,694 profiles from the normal kinship category and 9,695 profiles from the incest kinship category were correctly classified. On the other hand, 306 profiles that the model identified as cases of incest kinship and 305 profiles that the model classified as normal kinship were misclassified.

**FIGURE 3:**
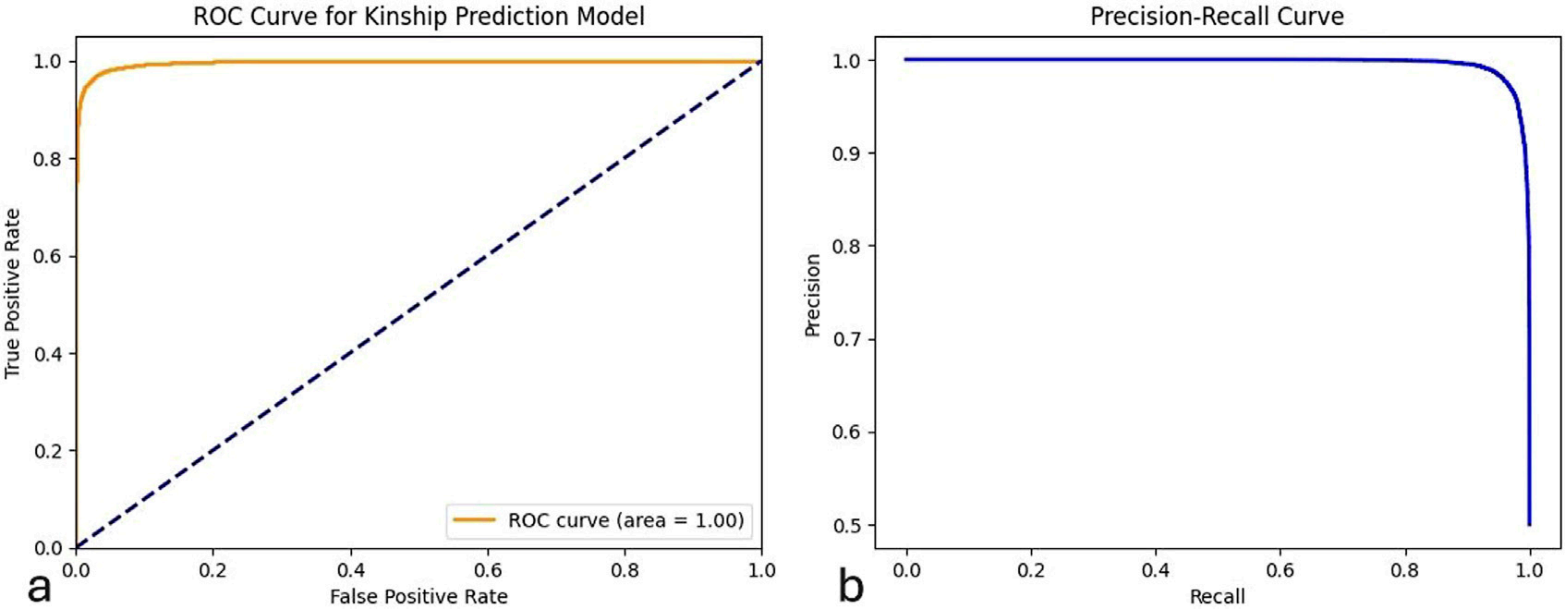
ROC and Precision-Recall curves for the general population model using 29 STR markers. **(a)** ROC curve showing the true positive rate against the false positive rate for the classification of normal versus incest kinship. **(b)** Precision-Recall curve indicating the trade-off between precision and recall for the same model.

The ROC AUC score of the model is presented in Figure 3a, and it quantifies the ability of the model to distinguish 99.62% between the two target classes. The balanced accuracy score, which takes into account model performance for both classes, was 96.94%. The Positive Predictive Value (PPV) was 96.94%, while the Negative Predictive Value (NPV) was 96.95%. The detection rate of the model, representing the percentage of true positive cases that were correctly identified, was 96.95%. Figure 3b illustrates the Precision-Recall Curve. The accuracy of the model based on the correct classification of positive cases as real positive was 96.94%, and the proportion of actual positive cases that are correctly identified by the model (recall) was 96.95%, also shown in Figure 3. The F1 score indicates the efficiency of the model with 96.95%.

Matthews’s correlation coefficient (MCC) was 93.89%, indicating a strong correlation between observed and predicted classifications. The specificity of the model was 96.94%, which means that it correctly identified 96.94% of all negative cases. The False Positive Rate (FPR) was 3.06%, while the False Negative Rate (FNR) was 3.05%. Finally, Cohen’s Kappa score was 93.89%, reflecting a significant degree of agreement between predicted and actual classifications.

### 3.4 Genetic markers and indicators of consanguinity

As the first indicator of consanguinity, the similarity between two STR profiles was examined by analyzing 29 markers, depending on the type of kinship. Table 2 presents the average values obtained from the analysis of 150,000 profile pairs for each of the examined categories.

**TABLE 2:** Average STR profile similarity measures across kinship categories based on 150,000 simulated comparisons.

Kinship category	Average profile similarity (%)
Parent-child (Normal Paternity)	59.42
Mother-child (Offspring from Father-Daughter Incest)	69.57
Sibling pair (Normal Sibling)	61.96
Mother-child (Offspring from Brother-Sister Incest)	69.53

Based on the obtained results, it is observed that, due to consanguinity, profile similarity increases by almost 10%, which is a consequence of a higher number of identical-by-descent (IBD) alleles between parents, leading to a reduction in genetic variability.

**FIGURE 4:**
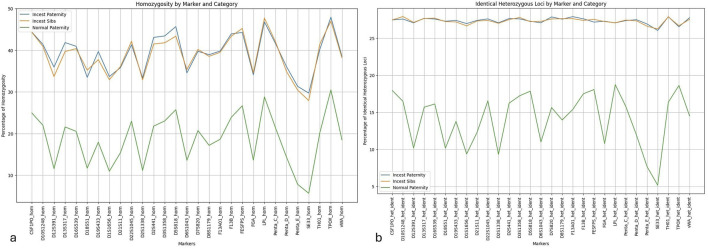
Distribution of homozygosity and identical heterozygous loci across 29 STR markers by kinship category. **(a)** Percentage of homozygosity per STR marker in three kinship categories: Incest Paternity, Incest Siblings, and Normal Paternity. Higher homozygosity is observed in incest categories, reflecting reduced genetic variability due to consanguinity. **(b)** Percentage of identical heterozygous loci shared between individuals in each category. Incest Paternity and Incest Siblings exhibit a greater proportion of identical heterozygous loci compared to Normal Paternity, indicating higher allelic sharing.

We further examined the percentage of homozygosity for 29 markers presented in Figure 4a, for three different categories: Normal paternity, Incest cases of parent-child inbreeding, and incest cases of brother-sister inbreeding. Within both groups of incest, an elevated rate of homozygosity (35%–45%) was observed, in line with the fact that higher rates of observed homozygosity were caused by inbreeding. On the other hand, for the category of normal paternity, the obtained values of homozygosity were between 10%–25%, in accordance with the greater genetic diversity within this category.

Examining the percentage share of identical heterozygous loci for all three categories (Figure 4b), a similar pattern was observed. More specifically, in cases of consanguinity, a higher number of identical loci shared by two profiles can be expected (25%–27%). In contrast, the category of normal paternity displays significantly lower ratios of identical loci, ranging from 10% to 20%, again proving that genetic diversity is greater within this particular category.

### 3.5 Model for the general population (21 Marker variant)

The model achieved an accuracy rating of 95%. The precision of the model was 95.28%, with a recall score of 94.69%, resulting in an F1 score of 94.98%. The ROC AUC was 99.01%, and the balanced accuracy was 95%. The MCC was 90.00%, and Cohen’s Kappa was 90.00%.

The confusion matrix for the general population is presented in Figure 2b. The model performance across classes is as follows: 9,531 profiles belonging to the normal kinship category and 9,469 profiles belonging to the incest kinship category were correctly classified. On the other hand, 469 profiles, that the model classified as cases of incest kinship, together with 531 profiles that the model classified as cases of normal kinship, were misclassified.

**FIGURE 5:**
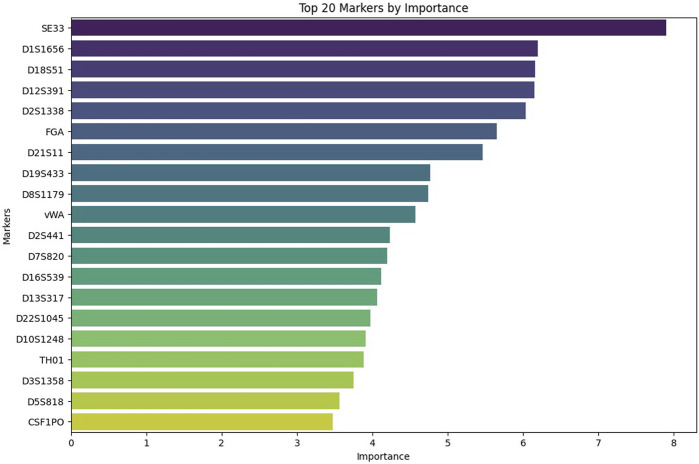
Top 20 STR markers ranked by their importance in the kinship prediction model. This bar chart shows the relative importance of the 20 most informative STR markers used by the CatBoost model trained on the general population dataset with 21 loci. Markers such as SE33, D1S1656, and D18S51 had the greatest influence on model predictions, while D3S1358, D5S818, and CSF1PO contributed the least.

The importance of individual markers for the accurate prediction is presented in Figure 5. This bar chart ranks the 20 most important genetic markers by order of significance for the accurate prediction of kinship. Markers such as SE33, D1S1656, and D18S51 hold the greatest importance for accurate prediction. On the other hand, loci such as TH01, D3S1358, D5S818, and CSF1PO, as well as the marker TPOX, which is not shown on the graph, were found to be the least informative for accurate prediction.

### 3.6 One-step mutation model for the general population (21 markers)

The model trained and tested on the general population database incorporating single-step mutation and utilising 21 markers as input variables, achieved an accuracy rating of 95.02%.

The confusion matrix is presented in Figure 2c. This particular variant of the model performed in the following way: 9,519 profiles belonging to the normal kinship category and 9,485 profiles belonging to the incest kinship category were correctly classified. On the other hand, 481 profiles, recognized as belonging to the category of incest kinship, together with 515 profiles, recognized as belonging to the category of normal kinship, were misclassified.

The model’s precision was 95.17%, with a recall of 94.85%, resulting in an F1 score of 95.01%. The ROC AUC was 99.01%, and the balanced accuracy rating was 95.02%. The MCC stood at 90.04%, and Cohen’s Kappa was 90.04%.

### 3.7 Performance metrics for the “offspring-only” model (21 markers)

This particular variant of the model was trained using only offspring profiles taken from the general population database, with 21 markers as input variables. The model achieved an overall accuracy rating of 90.37%.

The confusion matrix is presented in Figure 2d. The model’s performance is as follows: 16,316 profiles belonging to the normal kinship category and 15,315 profiles belonging to the incest kinship category were correctly classified. On the other hand, 1,184 profiles, recognized as belonging to the incest kinship category, and 2,185 profiles, recognized as belonging to the normal kinship category, were misclassified.

The model’s precision was 92.82%, with a recall of 87.51% and an F1 score of 90.09%. The ROC AUC was 96.34%, and the balanced accuracy was 90.37%. The MCC stood at 80.88%, and Cohen’s Kappa was 80.75%.

### 3.8 PCA analysis

As our multiclass prediction model, targeting not only incest detection, but also the potential sub-categories of incest, failed to achieve satisfactory performance metrics, we decided to apply Principal Component Analysis (PCA).

**FIGURE 6:**
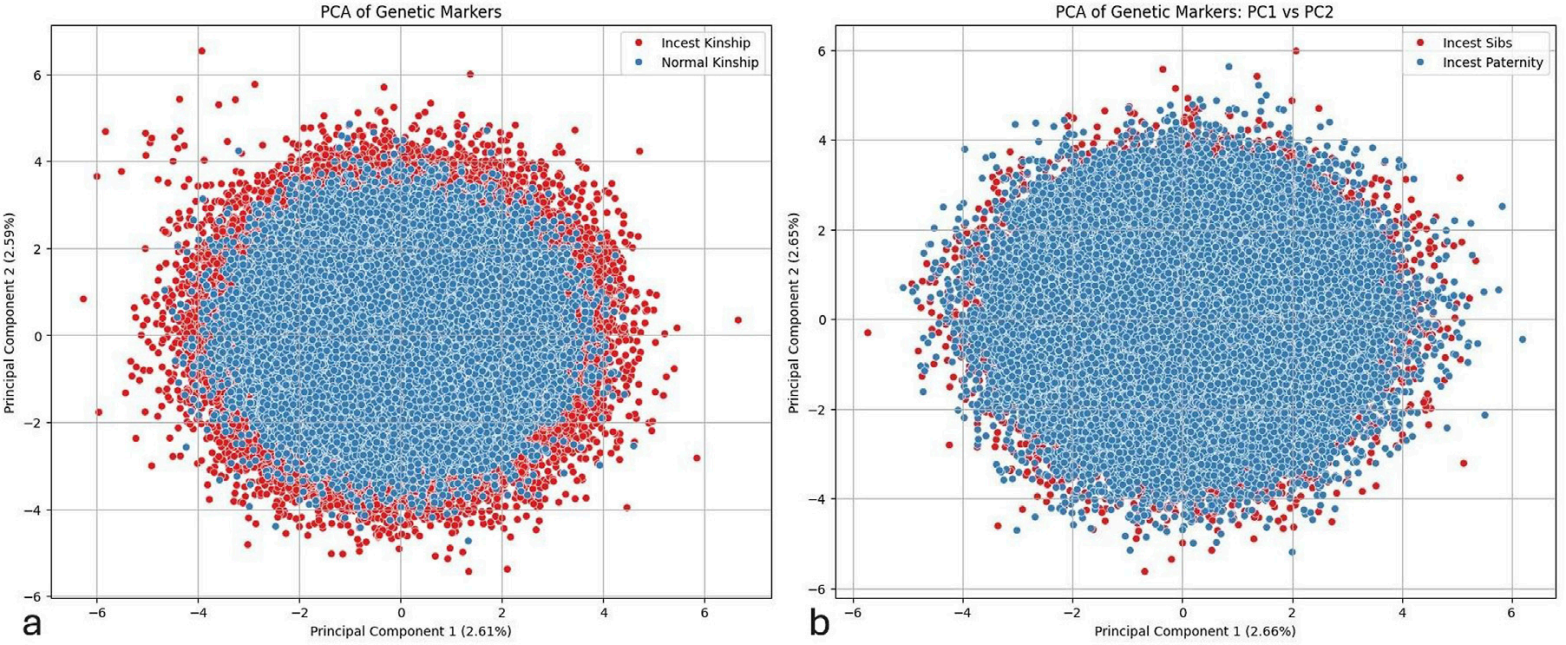
PCA plots of genetic marker distributions for kinship classification. **(a)** Principal Component Analysis (PCA) plot of Normal Kinship (blue) and Incest Kinship (red) instances using the first two principal components (PC1: 2.61%, PC2: 2.59%). A ring-like pattern is observed, with incest profiles forming a peripheral cluster around centrally positioned normal profiles. **(b)** PCA plot comparing Incest Sibs (red) and Incest Paternity (blue) profiles along PC1 (2.66%) and PC2 (2.65%). Incest Sibs show greater dispersion, while Incest Paternity instances form a denser core, indicating subtle genetic differences between the two incest subcategories.

The individual instances in Figure 6a are plotted along two axes corresponding to the two principal features of greatest importance for the differences between normal and incest kinship. A distinct structure can be observed on the scatter plot, with instances of incest kinship forming a ring around the central portion of the plot, occupied by instances of normal kinship. The first two components explain 2.61% and 2.59% of the variance, respectively. The clustering pattern suggests that genetic markers used in this study can differentiate between the two categories in question. Note, however, the existence of outliers from the normal paternity category that cross into the areas mostly populated by instances of incest kinship.

The scatter plot in Figure 6b shows the distribution of genetic markers for Incest Sibs (red) and Incest Paternity (blue) along the first two principal components. Similar to the previous plot, the first principal component (explaining 2.66% of the variance) is plotted on the x-axis, and the second principal component (explaining 2.65% of the variance) is on the y-axis. The plot indicates that instances of Incest Sibs are dispersed more around the periphery, whereas Incest Paternity profiles predominantly form a dense central core. This pattern suggests a slight but discernible differentiation between the two incest categories, with Incest Sibs exhibiting greater variation in their genetic markers compared to Incest Paternity.

### 3.9 The Saudi Arabia dataset - the initial and modified models

The initial model trained and tested on the dataset emulating the genetic structure of the Saudi Arabian population, using 21 markers as input variables, demonstrated an accuracy rating of 94.64%. The confusion matrix is presented in Figure 2e. This model correctly classified 9,533 profiles, as belonging to the normal paternity category, and 9,394 profiles, as belonging to the incest kinship category. The model misclassified 467 profiles, belonging to the incest kinship category, and 606 profiles, belonging to the normal paternity category.

The precision of the model was 95.26%, with a recall of 93.94% and an F1 score of 94.60%. The ROC AUC score was 98.85%. The balanced accuracy rating was 94.64%, with a PPV 95.26%, and a NPV of 94.02%. The detection rate was 93.94%.

The second model trained and tested on the modified version of the Saudi Arabia dataset with modified frequency values had the following performance metrics. The accuracy rating of the model was 94.93%. The confusion matrix is presented in Figure 2f. This model correctly classified 9,566 profiles, as belonging to the normal paternity category, and 9,421 profiles, as belonging to the incest kinship category. The model misclassified 434 profiles, belonging to the incest kinship category, and 579 profiles, belonging to the normal paternity category.

The precision of the model was 95.60%, with the recall 94.21%, indicating that the model correctly identified 94.21% of all actual positive cases of incest. The F1 score was 94.90%. The ROC AUC score was 98.94%, highlighting the model’s ability to distinguish between positive (incest) and negative (normal) classes. The balanced accuracy, which is the average of sensitivity for each class, was 94.93%, accounting for the imbalance between classes. PPV was 95.60% and NPV was 94.29%. The detection rate was 94.21%, the same as recall, representing the percentage of correctly identified actual positive cases.

## 4 Discussion

The main contribution of the study is the integration of machine learning into the detection of incestuous conception based on STR profiles. The study demonstrated that by using the described model, reliable detection of incest is possible, even when based solely on children’s profiles or in the presence of mutations. This represents an improvement over previously described approaches that require additional STR markers, trio analyses, as well as complex LR-based biostatistical calculations.

Across all tested variants, the model consistently demonstrated high classification accuracy: 96.94% for 29 markers, 95.00% using 21 markers, 95.02% under a one-step mutation model, and 90.37% in the offspring-only scenario. When applied to a dataset reflecting the Saudi Arabian population structure, the model achieved 94.64% accuracy, increasing to 94.93% when allele frequencies were modified. This confirms the advantages of using machine learning for detecting subtle patterns caused by inbreeding. It can be assumed that the observed errors in classification are the result of various factors, including: overlap of allele combinations between different classes, subtle or unexpressed homozygosity, a reduced number of markers, as well as the presence of profiles that manifest characteristics of both classes and were therefore incorrectly recognized by the model.

### 4.1 Comparison with previous studies

One of the first approaches in incest detection was based on the use of the Avuncular Index (AI) and the Incest Index (II). AI tests whether the uncle is the biological father, while II estimates the probability that the child’s father is a close relative of the mother, such as her father or brother. This approach enabled the detection of 3 cases of incest out of 1,500 analyzed cases, which indicates the limited sensitivity of the methodology itself. The AI values for the uncle were found to be significantly higher than those obtained for a random male. For the uncle, this value was 7.7, while for a random man it was only 0.26. The significance of the research is also reflected in the fact that the authors found that identical or homozygous HLA haplotypes between the mother and child can indicate incest (Morris et al., 1988).

A study that calculated the combined incest index (CII) from mother-child profiles using 18 STR loci was able to produce diagnostic CII values in only 2 of 5 suspected incest cases. The authors note that 18 STR markers are not sufficient to accurately distinguish these relationships. According to their estimate, the analysis of 33 independent STR loci is necessary to achieve an accuracy of 97.5% (Wenk, 2008).

The probability of paternity in incest cases is lower compared to paternity in cases of random mating. The reason for this lies in the fact that the allele that is present in the mother can be found in both the alleged father and the child. When the child shares an allele with the father at a heterozygous locus that is not present in the mother, the probability of paternity increases (Minakata et al., 1996).

The methodology based on different combinations of genotypes of parents and offspring allows estimation of the probability based on the distribution of alleles between the mother, the supposed father (who may be a brother), and the offspring. With this approach, incest can be reliably detected if a sufficient number of markers are used, but additional data and analyses are required for a reliable result (Tamura et al., 2000).

### 4.2 Influence of inbreeding on genetic markers

Based on the STR profile similarity observed in this study, it was found that the average similarity between parent and child is 59.42%, while for siblings it is slightly higher at 61.96%, indicating that in both categories, corresponding to normal relations, the profile similarity is about 60%. Due to inbreeding between a father and his biological child, the similarity between the child’s profile in incest paternity and the mother’s profile is on average 69.57%, which is close to 69.53%, representing the similarity between the mother’s profile and a sibling’s incest child. The same percentage of approximately 69% was also observed between the mother and child from a brother-sister relationship based on multilocus probing (Tamura et al., 2000). It can be concluded that the similarity between parent and child profiles is almost 10% higher due to inbreeding compared to random mating profiles.

Increased homozygosity in the child’s profile can be an indicator of incest, as presented in a study that cited a case of a 21-year-old woman with severe mental and physical disabilities, where the child’s profile showed homozygosity on 8 out of 15 analyzed loci. This suspicion resulted in further profiling of the male relatives, confirming that the case involved a brother-sister incestuous relationship (Robino et al., 2006). However, homozygosity is not always a definitive indicator of incest, as seen in the analysis of presented STR profiles, where homozygosity in the child was observed at 5 of the 22 analyzed loci, one locus more than the number of homozygous loci in the mother (Canturk et al., 2016).

It has also been confirmed that due to consanguinity, the rate of identical heterozygous loci between the mother and child is elevated, which further indicates reduced genetic diversity and inherited patterns resulting from incest. These patterns are reflected in a higher degree of identical allele combinations at heterozygous loci.

The examined genetic indicators of incest are a consequence of an increased presence of identical-by-descent (IBD) alleles, and consequently, a reduction in genetic variability that may manifest in offspring (Stoffel et al., 2021). Importantly, all factors (profile similarity, homozygosity, and identical heterozygous loci) should be considered together rather than separately, as one of these factors may be “masked.”

On the other hand, these findings represent universal principles of allele distribution patterns, as well as their dependence on the tested population. For example, if these markers were analyzed in an isolated population, the similarity between mother and child profiles would certainly be higher compared to the values observed in the general population, but still lower than those observed as a result of incest in such populations. This emphasizes that analysts must be familiar with the ancestry of the examined individuals in order to assess whether their genetic heritage could influence prediction.

Although the PCA analysis detected sufficient levels of variations within both categories of profiles, high similarity rates between incestuous profiles and the overlap of data points belonging to the previously mentioned categories of incest were major barriers that prevented the successful deployment of an effective multiclass classification model (See Supplementary Figure S1 Multiclass prediction). As a result, we opted to create a binary model, which was trained using profiles belonging to both categories of incest which were collapsed into a single, overarching category. The resulting basic model, which was based on the display of two STR profiles parent-child, without additional features such as profile similarity, child homozygosity and identical markers between mother and child, always yielded better performance metrics. However, it should be noted that the performed PCA analysis revealed clustering patterns that might be handled more effectively by more sophisticated algorithms, which will, in turn, allow us to overcome the multiclass prediction problem described above.

### 4.3 Preliminary study and STRIDER frequencies

In the preliminary study profile generation was done based on STRIDER frequencies for all loci and detected alleles (STRIDER, 2024). The model trained and tested using this data achieved a classification accuracy of almost 98% for all four categories, allowing the model not only to correctly predict cases of incest but also to correctly classify the category of incest. Subsequent testing of this variant of the model on a database using frequencies obtained from other populations used in this study failed to yield equivalent results. The subsequent analysis revealed that the initial similarity of the parental profiles based on STRIDER frequencies was significantly lower (8–15 percent) compared to frequencies present in other populations. Due to the large number of allele combinations at each locus, the model trained on STRIDER frequencies had no difficulty in distinguishing between incestuous and normal kinship and was even able to correctly categorise incestuous kinship into the subcategories mentioned previously. Of course, after the irregularities were discovered, these results were excluded from the study as the profiles based on STRIDER frequencies are not realistic in the context of actual parental profiles. Nonetheless, the findings obtained by the preliminary study still warrant discussion.

### 4.4 Potential benefits of machine learning in forensic genetics

We consider this study to be indicative of the potential benefits that machine learning can bring to the field of forensic genetics by offering a reliable method of detecting not only incest, but possibly also other complex models of kinship. This approach has much to recommend it - the creation of synthetic profiles and their further pairing to create composite profiles belonging to different categories of kinship allows researchers to sidestep a major hurdle in forensic science that usually manifests itself as an inability to examine all possible inheritance patterns that occur within different kinship categories. As the current trend in forensic genetics is to increase the number of analysed loci, it can be expected that by implementing additional loci, we would be able to improve the efficiency of the model itself.

### 4.5 Performance of CatBoost algorithm

Of all the gradient boosting algorithms that were tested within the scope of this paper, CatBoost exhibited the best performance metrics using both out-of-the-box and optimised parameters. It is interesting to note that even the implementation of one-step mutations, which are encountered in practice and which in the current analyses of real scenarios result in lower accuracy metrics, did not impact the performance metrics of the CatBoost model. One possible explanation for this behaviour can be found in the fact that the introduction of one-step mutations into the database introduced additional variation that was informative for the model, allowing the model to preserve its performance by leveraging the newly added information to better differentiate between categories.

Previous studies investigating effective methods for incest detection commonly included a caveat noting that it was unknown how the proposed solutions would behave in cases in which the individuals under investigation hailed from isolated populations. This was the main impetus behind our drive to implement our model on realistic datasets with actual frequencies obtained from the Saudi Arabian population (as well the modified version of the same dataset). It is interesting to note that the model trained on the modified dataset containing fewer alleles, exhibited a slight accuracy increase compared to the model trained and tested on the unmodified Saudi Arabia dataset. One possible explanation for this behaviour is that the exclusion of low frequency alleles from the dataset also reduced the noise that was otherwise present when these alleles were included in the dataset.

One surprising outcome was the model’s exceptional ability to accurately determine whether an offspring profile originated from a normal or incestuous union based solely on the child’s STR markers. The model trained and tested using solely unique offspring profiles had an accuracy rating of almost 90%. The addition of a mother profile to the offspring profile raised accuracy by only 5%. This constitutes clear proof that offspring profiles are informative by themselves.

It is likely that the analysis of a larger number of polymorphisms would enable the model to achieve equal if not better performance. The obtained results would certainly be useful in the field of clinical medicine, allowing future researchers to probe further into recessive diseases that can occur as a result of inbreeding.

### 4.6 Advantages of the model

In the context of a step-by-step analysis for detecting incestuous relationships, the model developed in this study would be most effective as a preliminary screening tool. The following workflow is envisaged: (1) STR profiling of underage pregnant individuals and their offspring, or fetal material in cases of abortion; (2); application of the classification model to detect potential incestuous conception; (3) identification of potential incest cases based on genetic deviations from expected patterns; (4) conducting additional confirmatory analyses, which may include profiling of the alleged father or brother, extended genetic testing, or medical examinations, depending on the context of the case.

One significant advantage of this model is that its prediction does not require the profiling of the father or the siblings, especially in cases in which the subjects are not available. Given the high accuracy of the model and the low percentage of false positives and false negatives, it can be used to initially signal potential incest, especially in cases involving minors, where suspicious pregnancies can be investigated using solely the STR profiles of the mother and the child.

This approach allows the model to independently learn incest patterns based on the presented databases. As a result, the model is not “rigidly” defined by assumptions about the distribution of alleles between parents and offspring, which is the basis of traditional probability calculations for the confirmation of incest.

### 4.7 Limitations of the study

It is important to note that a positive prediction generated by the model does not confirm incest, but indicates the need for further action. Additional analyses would be necessary to confirm incest and identify the perpetrator. Even though the model performed well, the possibility of error still exists. It is important to be aware of the nature of the population from which an established profile is sourced so that an adequate model can be applied. To illustrate, it is highly likely that the profile of a child hailing from a normal crossbreeding scenario occurring with an isolated population would be recognized as a false positive by a model trained on general population data due to decreased allele variety and higher homozygosity levels. On the other hand, it is also highly likely that the profile of a child born from an incestuous union occurring within the framework of a general population would be recognized as belonging to the category of normal paternity by a model trained on isolated population data, producing a false negative. Our model was trained to detect incest resulting from father-child and brother-sister relationships, however we do not know how the model would perform in incestuous cases with other male relatives. Future work could explore testing the model with other relatives or developing a more complex model for broader incest detection.

## 5 Conclusion

The study demonstrates the potential uses of machine learning in the field of forensic genetics when analysing complex types of kinship, such as incest. With the *in silico* study, we managed to overcome the problem of insufficient profile quantity for the training and the testing of the model. We successfully made predictions based on unique STR profiles and validated the model on allele frequencies from different populations. It can be concluded that the structure of the population itself does not play a key role in the model’s performance, allowing its implementation to different populations. Additionally, the model presented here does not require the initial profiling of the father and can be used as a tool to signal potential cases of incest.

## Data Availability

The datasets presented in this study can be found in online repositories. The names of the repository/repositories and accession number(s) can be found below: https://zenodo.org/records/13287992, https://doi.org/10.5281/zenodo.13287991.
