# Supplementary figures and images for: From genetic data to kinship clarity: employing machine learning for detecting incestuous relations
Source: Front Genet. 2025 Jun 2;16:1578581. doi: 10.3389/fgene.2025.1578581 (PMC12171372; doi:10.3389/fgene.2025.1578581)

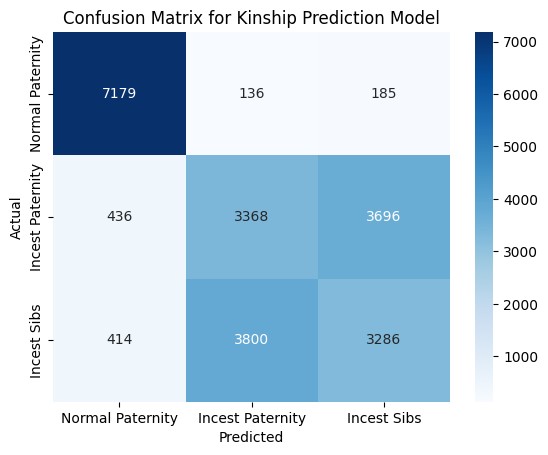

Supplement: Supplementary file 1 [file Image1.jpeg]
